# Peyronie's Disease: Evolving Surgical Management and the Role of Phosphodiesterase 5 Inhibitors

**DOI:** 10.1100/tsw.2009.101

**Published:** 2009-08-31

**Authors:** Tariq F. Al-Shaiji, Gerald B. Brock

**Affiliations:** Division of Urology, Department of Surgery, University of Western Ontario, London, ON, Canada

**Keywords:** Peyronie’s disease, surgical therapy, optimizing outcome, phosphodiesterase 5 inhibitors, future directions

## Abstract

Peyronie's disease (PD) is a fibrotic disorder of the tunica albuginea of the penis. It is characterized by different degrees of penile curvature and sexual dysfunction. Several medical treatments have been employed to manage the disorder, with variable success rates. Surgical therapy is reserved for patients with severe penile deformity that fails to improve with medical treatment and impedes coital function. The advantages and disadvantages of various surgical approaches have long been debated. Herein, we describe the evolving surgical techniques for PD using knowledge obtained from the contemporary literature. In addition, we discuss the emerging data regarding the role of phosphodiesterase 5 inhibitors in the management of PD.

## INTRODUCTION

Peyronie's disease (PD) was first described by Francois de la Peyronie (1678–1747), who reported the first series of patients with "rosary beads of scar tissue" causing curvature of the penis[1]. It is characterized by scarring of the tunica albuginea, which eventually loses elasticity[2]. The development of the disease is best thought of as an inappropriate response to wound healing resulting in scar formation, deformity, and, not infrequently, diminished rigidity[3]. The condition is invariably associated with penile length reduction and can have a major impact on quality of life, with significant psychological effects[4]. PD patients display a higher risk of depression, lowered self-esteem, and difficulties with relationships[5]. It has been postulated that PD results from repetitive minor trauma to the tunica albuginea of the penis during intercourse or sexual activity[6].

Schwarzer et al. reported that the condition afflicts 3.2% of adult men, with an average age of onset at 57 years[7]. Furthermore, Tunuguntla[8] nicely summarized the epidemiology of PD as the following:

- Occurs in 0.38–3% of the adult male population (40–60 years).
- Occurs in 6.5% of men older than 70 years.
- Occurs in 1.5% in the 30- to 39-year age group

Recent emerging data suggest that the prevalence of PD is similar to that of diabetes mellitus and renal calculi[9].

From a patient's prospective, the main presenting symptoms of PD include penile pain on erection, erectile curvature, painful intercourse, erectile dysfunction (ED), and an indurated penile plaque[1]. The most common location for the plaque is on the dorsal midline of the penis, resulting in an upward curvature[8], whereas lateral or ventral plaques are less common[5] (Fig. 1). Dorsal and ventral plaques may connect through septal extensions to form an I-beam, causing shortening without angulation[8,10] (Fig. 1). Penile deformity is the most common first symptom of PD, as reported by Pryor and Ralph[11]. However, in a large-scale series, Cakan et al. showed that the most common presenting symptom was ED[12]. Usta et al. showed that ED is strongly associated with PD ranging from 20 to 54%[13]. The penile deformity may also prevent vaginal penetration or satisfying sexual intercourse[14]. One of the more common reasons that patients seek treatment involves partner discomfort during intercourse, which is associated with penile curvature[15]. There are emerging data suggesting that men with PD are depressed and their quality of life is impaired[5]. Using validated instruments, Nelson et al. examined 92 men presenting for PD evaluation and demonstrated that 48% of the cohort had clinically meaningful depression that would warrant medical evaluation[16]. Painful erections and a palpable penile plaque are usually the clinical hallmarks of the acute phase of PD (12–18 months), whereas the chronic phase is marked by stabilization of the penile deformity, hardening of the plaque, and ED[17]. Egydio defined disease stability as at least 1 year of disease, and at least 6 months of nonprogression or regression of penile deformity and/or plaque, and absence of pain[2]. Twenty to 50% of patients with PD may experience spontaneous resolution[18], however, Mulhall et al. reported that untreated PD resolves in only 12% of men, with 40–48% of men demonstrating worsening of curvature at 12 months, and stable curvature in the remaining men[19]. Nevertheless, Hellstrom and Bivalacqua stated that the standard of care for newly diagnosed PD involves a conservative approach (using oral agents, intralesional therapies, or both) for the first year[17]. It is important to note that observation (nontreatment) alone is a viable option as well[20]. Many PD patients may not experience any symptoms and reassurance that the palpable lesion is not cancer is all that is needed[8]. Surgical management is indicated on failure of conservative or local therapies[2,21,22]. Authorities in the field of PD have discussed several criteria essential before surgical intervention is contemplated, which can be summarized as[14,17,23,24]:

1. Severe curvature, narrowing, or indentation of more than 12 months duration
2. PD stability for at least 3 months
3. Curvature that precludes sexual intercourse
4. Severe penile shortening

Contraindications to surgery — in a patient who presents with[25,26]:

1. Active disease
2. Progressive disease
3. Mild curvature not precluding penetrative intercourse
4. When there is plaque without curvature (preventive plaque resection)

## PREOPERATIVE ASSESSMENT AND SURGICAL OPTIONS

All surgical approaches must be individualized to meet the patient's penile deformity, clinical manifestations, and expectations[2,14]. Variations in the nature and severity of penile deformity and erectile capacity indicate that there is no single standard or best surgical therapy for PD[27]. Patient evaluation and selection before any given surgical intervention is of paramount importance, and this is important not only to plan therapy, but also for medico-legal reasons[8]. Bokarica et al. recommend basing the surgical technique selection on penile length and curvature degree[28]. They further state that

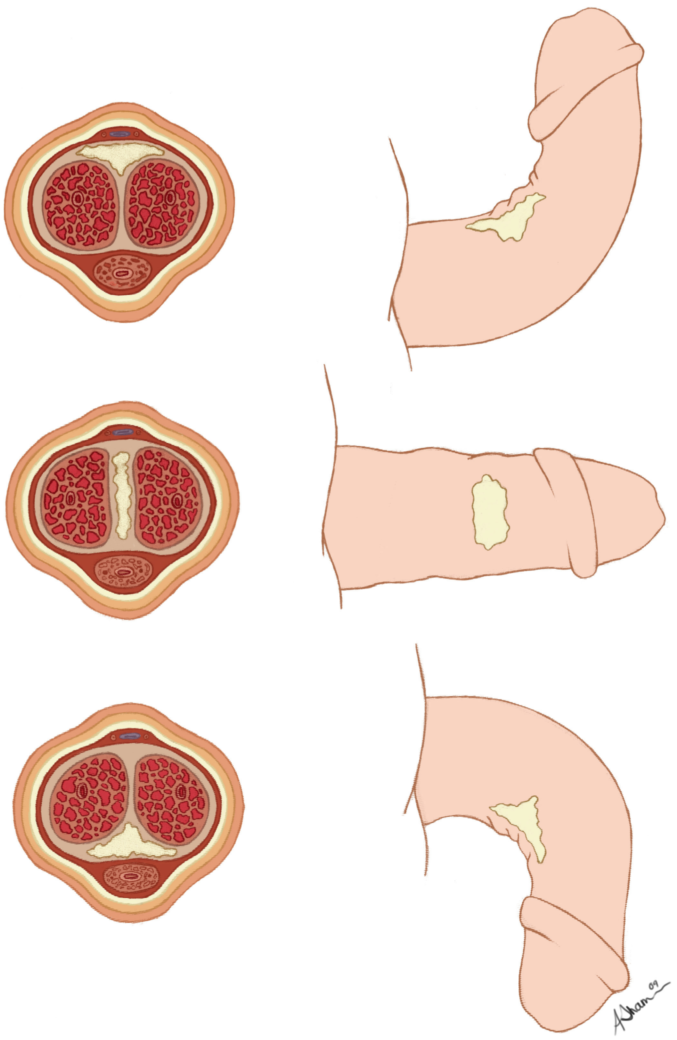

**FIGURE 1.** Locations of PD plaque. Dorsal midline plaque results in an upward curvature, whereas ventral midline plaque results in a downward curvature. Dorsal and ventral plaques may connect through septal extensions to form an I-beam, causing penile shortening without angulation.

the satisfaction of most patients, in spite of significant penile length loss, was largely due to proper patient selection and preoperative information. It is essential to document, through patient reports, pictures, Rigiscan, or Doppler ultrasound, that patients are able to have satisfactory erectile function and vaginal penetration[1]. Most authorities recommend a detailed evaluation of penile vascular status and erectile function, as the presence of one or both dictate subsequent surgical approach[17,24,29]. Published reports indicate that between 30 and 80% of men may have ED associated with PD, and up to 50% may have ED before they develop PD[30,31]. Existing studies have demonstrated repeatedly that the ED found in men with PD is most commonly due to vascular insufficiency similar to the etiology found in the general public with ED alone in which arterial insufficiency predominates as the primary etiology with or without venous insufficiency[32,33,34].

Erectile function cannot be accurately estimated from patient-provided photographs alone, and is best established using a patient history and an instrument such as the International Index of Erectile Function-5 (IIEF-5)[35]. Penile vascular abnormalities are commonly associated with PD[24]. In a retrospective study, Kendirci et al. correlated vascular status to type of penile deformity, demonstrating a relationship between type of curvature and penile hemodynamics[36]. Brock et al. stated that hemodynamic assessment with duplex ultrasound is appropriate and should be undertaken before any surgical intervention[37]. The test allows for a more precise definition of the vascular etiology of ED and determines erectile response to an injected vasodilator. In addition, it is possible to assess the location of the dorsal penile, artery-perforating vessels that may be interrupted during elevation of the vascular bundle that may exacerbate ED postoperatively in those with borderline erectile capacity[27]. In a study by Kadioglu et al.[38], color Doppler ultrasonography showed penile vascular abnormalities in 76.5% of patients with PD. Veno-occlusive dysfunction was noticed in 40% of men with normal erection, whereas mixed (arterial + venous) vascular disease was diagnosed in 10%. Although corporeal veno-occlusive dysfunction has been considered the primary vascular etiology of ED associated with PD, arterial insufficiency is a major contributor, which is best detected before definitive therapy as well[32]. Finally, before any surgery is undertaken, all patients must undergo informed consent, discussing the potential risks of postoperative ED, penile shortening, distal penile softening, future plaque formation, new or recurrent deformity, and the possibility for a sensory deficit[20,39]. It is not uncommon that patients with unrealistic expectations will not be satisfied with any surgery and are not necessarily considered good candidates for surgical intervention[17,29,40]. The overall quoted success rates of PD operations have ranged between 64 and 100%[41,42,43].

The surgical approaches to PD can be divided into two broad categories: correction of the penile deformity (shortening or lengthening procedures) or the insertion of a penile prosthesis (Table 1). The various operations are discussed below.

**TABLE 1**
**Surgical Approaches to PD**

| Category | Techniques | Indications |
|---|---|---|
| Penile shortening procedures (shortening of the convex side of the tunica albuginea) | Plication or wedge resection<br>Nesbit procedure<br>Modified Nesbit<br>Plication variations and corporoplasty | Subjective full erectile capacity and/or objective full erectile capacity[27]<br>Adequate penile length (>13 cm)[5,20,27]<br>Mild curvature (<60 degrees)[1,27]<br>Minimal plaque[1]<br>No hourglass deformity or hinge effect[27] (Fig. 2) |
| Penile lengthening procedures (lengthening of the concave side of the tunica albuginea) | Grafting procedures<br>Plaque incision and grafting<br>Plaque excision and grafting<br>Plaque incision/excision and vascularized flap | Subjective full erectile capacity and/or objective full erectile capacity[27]<br>Small/short penis[5,44]<br>Curvature (>60 degrees)[27,44]<br>Proximal curvature[24]<br>Presence of hourglass deformity or hinge effect[5,27,44] |
| Penile prosthesis implantation | Penile prosthesis<br>Penile prosthesis + manual modeling (molding)<br>Penile prosthesis + Nesbit/plication<br>Penile prosthesis + incision/ excision with grafting | Concomitant ED refractory to pharmacologic therapy[45]<br>Vascular insufficiency[24] |

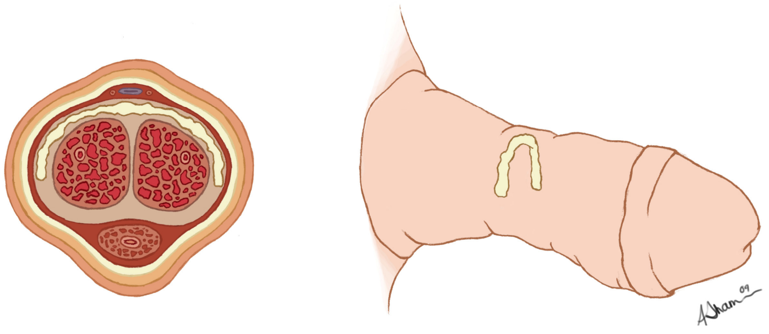

**FIGURE 2.** Circumferential scar causing bottlenecking/hourglass deformity.

## Penile Shortening Procedures

Shortening procedures are generally considered to be easy to perform, provided that the patient is well selected[24]. For men reporting good erectile function, adequate penile length, and curvature without narrowing abnormality (Table 1), plication surgery is an excellent surgical option. These procedures are associated with penile length reduction as well as diffuse elasticity decrease of the tunica in the longer side, which may further add to penile length loss (from PD and from surgery)[2]. In general, the stretched flaccid length of the penis is the expected length after the plication procedure, and this must be demonstrated to the patient before surgery and discussed in the informed consent[46]. Preoperative counseling usually minimizes the patient's concern regarding penile length and nodules[14].

In 1965, Nesbit[47] first described the correction of congenital penile curvature in which an ellipse of tunica albuginea was excised from the unaffected side of the penis before plication at the most prominent portion of the curvature. In 1979, Pryor and Fitzpatrick subsequently reintroduced Nesbit's technique for the surgical correction of PD[48]. Based on the degree of curvature, more than one ellipsoid may need to be excised in order to correct the deformity completely. Closure of the defect is made with a nonabsorbable atraumatic suture, with burying of the suture knots. Ralph et al. reported their experience with the Nesbit procedure on 359 PD patients over 16 years and showed a satisfaction rate of 82% utilizing this technique[49]. Complications commonly reported with the Nesbit procedure include penile shortening, ED, penile hematoma, urethral injury, glans numbness, phimosis, penile narrowing and indentation, herniation, palpable suture "knots", and suture granuloma[25,26,49]. Van der Horst et al.[50] discussed the importance of selecting appropriate sutures for plication procedures in order to prevent the development of granuloma and pain, which can impair the patient's quality of life. It was shown that polytetrafluoroethylene sutures resulted in only 13% of the patients complaining of postoperative pain vs. 52% with polypropylene sutures.

A number of modifications of the Nesbit technique have been described. Rehman et al. reported a modification with partial thickness shaving instead of conventional excision of a wedge of tunica albuginea[51]. This modified procedure was employed in 32 patients with congenital penile curvature and PD with satisfaction rates of 100 and 78%, respectively. The technique minimizes intraoperative bleeding, obviates cavernous tissue damage, and improves adhesion of plicated tunical layers.

Lemberger et al.[52], and more recently Yachia[53], proposed their modification of the Nesbit procedure in which longitudinal incisions made in the tunica albuginea are closed horizontally in a Heineke-Mikulicz manner. These authors reported satisfaction rates ranging between 79 and 95% with their technique. Giammusso et al.[54] published a modified Yachia procedure consisting of resecting the deep dorsal vein, and performing a longitudinal incision and transversal suture with absorbable suture on the venous bed, achieving 100% rectification without the need to mobilize the neurovascular bundle. When absorbable sutures are used, this may prevent pain and definitive palpability of the stitch.

Another modification was described by Essed and Schroeder in 1985[55]. Nonabsorbable sutures are placed on the convex side of the tunica albuginea without excision/incision. Friedrich et al. performed a retrospective analysis assessing the long-term functional results and quality of life of patients after undergoing the Essed-Schroeder procedure, and documented the cosmetic and functional results to be sufficient in 81% of patients[56]. Erpenbach et al used the plication technique and reported a 96% satisfaction rate[57].

Recently, Gholami and Lue introduced a novel modification of the penile plication approach[58]. The technique is referred to as the 16- or 24-dot plication procedure, and consists of the placement of multiple, paired plication sutures over a long span on the penis in a nontension manner without any incision/excision. The multiple sutures can easily be adjusted throughout the surgery before being tied, so that a straight penis can be accurately created. This procedure is carried out under local anesthesia and an artificial erection is obtained using corpus cavernosum injection of papaverine. The authors reported their experience with this technique in 132 cases (116 Peyronie's patients and 16 with congenital penile curvature) after a mean follow-up of 2.6 years. Postoperatively, 93% of the 132 patients reported straight erections, while 7% complained of slight curvature, and 4% complained of decreased erectile function. Penile shortening was noted in 41%. This approach has the following advantages: minimal risk of ED, minimal risk of neurovascular damage, precise placement of sutures, and the fact that the surgery can be performed with local anesthesia with or without sedation on an outpatient basis[14,26]. On the other hand, the disadvantages are: penile shortening, palpable nodules from permanent sutures, and, occasionally, prolonged pain from sutures[14].

## Penile Lengthening Procedures

Lengthening procedures are generally indicated in patients with severe or complex deformities, extensive plaque(s), and short penises (Table 1). The procedures involve the use of reconstructive techniques to lengthen the concave size by either incising or excising the plaque and then covering the defect with graft material.

The graft materials are divided into three broad categories: autologous tissue, allograft or xenograft materials, and synthetic grafts (Table 2). Several factors dictate the choice of the graft material, including availability, cost, efficacy, safety, type of deformity, patient preference, and surgeon experience[59]. An ideal graft should be affordable, packaged and readily available in a variety of sizes, strong yet compliant (but not so compliant that aneurismal dilatation is possible), technically easy to use and suture, carry a low risk for infection and antigenicity, and cause minimal tissue reaction and patient morbidity[1,26,60]. It is critical to ensure that usage of any given material would still allow the goals of surgical repair in PD (including correction of curvature, maintenance of presurgical length, and postoperative patient satisfaction) to be achieved[61]. Complications associated with the use of grafts include ED, numbness of the glans and penile shaft, hematoma, wound infection, graft contraction, urethral injury, and urinary retention[20]. Some authors have suggested a possible role for concurrent prophylactic ligation of the dorsal vein in patients undergoing grafting in order to decrease the chance of postoperative ED, since studies with penile duplex Doppler have revealed significant venous leakage in some patients after dermal graft placement[20,62]. One complication associated with synthetic grafts is the substantial postoperative inflammation around the grafted site with subsequent recapitulation of the plaque and possible further curvature and, thus, even removal of these materials at a later date frequently produces further penile shortening and disability[1].

**TABLE 2**
**Graft Materials used to Cover Tunica Albuginea Defects[20,26,61]**

| Category | Material |
|---|---|
| Autografts | |
| Autogenous tissue from the patient's body, harvested by the surgeon at the time of surgery | Dermis |
| | Vein (saphenous, deep dorsal) |
| | Penile crura |
| | Dura mater |
| | Tunica vaginalis |
| | Fascia lata |
| Allografts/xenografts | |
| Materials harvested from another person or species | Cadaveric pericardium (human, bovine) |
| Tutoplast® processed allografts | Porcine small intestinal submucosa (SIS) |
| | Dermal allograft |
| Synthetic grafts | Polytetrafluoroethylene (Teflon®) |
| | Dacron® mesh |
| | Silastic® |
| | Gore-Tex® |

## *Excisional Procedures*

In 1947, Lowsley was first to publish a significant series of patients with PD treated by plaque excision and grafting (see [63]). The author reported a 66% success rate using a fat graft. The best results were found in patients with dorsal curvature and younger age. It was not until 1974 that Devine and Horton popularized excision of the entire plaque and use of a dermal graft[64]. They reported a series of 12 patients with PD and success was seen in up to 70% of men treated. Dermal grafts demonstrate higher tensile strength, yet maintain the compliance of skin when compared with fat. Sampiao et al. modified the excisional procedure by using an H-shaped incision to incorporate partial plaque excision and to allow for expansion of the diseased region of the tunica albuginea[65]. Although there has been some reported success, especially in young and potent patients, authors noted less than satisfactory results with dermal grafts, mainly because of graft retraction and high ED rates postoperatively[43]. In addition to free fat pad and dermal grafts, grafts have been harvested from rectus muscle aponeurosis, tunica vaginalis, temporalis fascia, saphenous vein, fascia lata, and a variety of other autogenous materials[66]. The primary advantages of autologous materials are excellent natural tissue incorporation into the tunical defect and the absence of additional costs; however, on the other hand, tissue harvesting may cause scarring on the harvest site, additional morbidity to the patient, and increased operative time[2,26]. The cadaveric pericardium has been described by a number of authors. Hellstrom and Reddy reported the use of cadaveric pericardium to graft the cavernosal defect in a modified Horton-Devine in a series of 11 patients grafted with pericardium and with a mean follow-up of 14 months[29]. Overall results were promising, with successful resumption of coitus in all patients and resolution of curvature in nine patients. Of note, three of the 11 patients had simultaneous penile prosthesis implantation for documented ED, and the use of cadaveric pericardium successfully reconstructed the corpus cavernosum in these individuals. In another modified Horton-Devine procedure, Chun et al. compared the results of plaque incision/excision and grafting with either dermal or cadaveric pericardial graft techniques[67]. The cadaveric pericardium was found to produce functional results and patient satisfaction similar to a dermal graft, but with a decrease in operative time of approximately 20 min. Erectile function was also maintained in both groups of patients in whom preoperative erectile function was reasonably intact. Usta et al.[68], as well, reported on long-term results of various surgical treatments for PD

with a mean follow-up of 21.9 months. Complete penile straightening was noted in 79% of patients who underwent penile plaque incision/excision and grafting with cadaveric pericardial graft material. Recently, Levine and Estrada retrospectively reviewed their experience with 40 men with PD who underwent penile straightening with partial plaque excision and grafting using human cadaveric pericardia from January 1999 to January 2003. The investigators reported 98% success in penile straightening, 95% success in intercourse, 70% with unaided full erections, and 30% with ED requiring pharmacological assistance. No major complication or graft-related adverse events were reported[69]. In the report by Usta et al., the incidence of postoperative ED increased when the size of the cadaveric pericardial graft was larger than 5 cm[68]. Using cadaveric pericardial graft material carries the following advantages: autologous graft harvest site is not necessary, shorter operative time, and lower patient morbidity. In addition, it also has the ability to expand multidirectionally, has excellent tensile strength, and is a relatively thin tissue[14,20].

Recently, in a rat model, Leungwattanakij et al. evaluated the intermediate and long-term efficacy of different grafting materials for tunica albuginea substitution after plaque excision for PD[70]. The authors applied a wedge excision of tunica albuginea to 100 male Sprague-Dawley rats and replaced the defect with either cadaveric pericardial, dermis, vein, or Gore-Tex® grafts. After 6 months, erectile function, assessed by cavernosal nerve stimulation, did not differ significantly in any of the groups. The histological assessment of penile cross-sections showed minimal fibrosis surrounding the patch in the dermal and vein grafts, and moderate to severe fibrosis in the Gore-Tex graft at 4 and 6 months. In the pericardial graft, there was a moderate degree of fibrosis at 4 months, with only minimal fibrosis at 6 months. While the ultimate fate of cadaveric pericardial allografts used for urological reconstructive procedures remains unknown, their use in neurosurgery as a substitute for dura mater has been widely reported. and reports on safety, long-term durability, and lack of antigenicity are emerging[1].

In 2004, Darewicz et al. published an alternative approach in which intracavernosal dorsal plaque excision was performed without grafting on 16 men[71]. The operation consisted of incising the corpus cavernosum parallel to the plaque and away from the neurovascular bundles, and through this incision, removing the plaque from the inside without incising or replacing the underlying tunica albuginea, which is then closed. Follow-up examinations made after 3, 6, and 12 months revealed normal, painless erection in all the patients. In two subjects, intercourse was impaired only by persistent penile curvature of over 20 degrees. All patients had significant improvements in IIEF-5 score.

Despite what has been mentioned so far, plaque excision should not be recommended as a standard first-line approach for most patients[72]. PD is considered to be a pan-tunical condition and excision may, therefore, have deleterious effects on the cavernosal function and veno-occlusive mechanism of the penis[73]. Furthermore, a major drawback to excisional procedures is the fact that not all patients have palpable plaques at the time of surgery (30%[74]). In the case of multifocal plaques, the issue is which of them should be excised; the tunica albuginea may be injured diffusely, and not plaque restricted, so that removal and grafting may not be sufficient to correct the curvature and restore length properly, requiring the addition of other relaxing incisions[2]. Several reports on excisional procedures using different grafting materials have shown variable outcomes, which may be associated with excision itself rather than grafting. Egydio et al. reported some discouraging results with cadaveric bovine pericardia associated with plaque excision, however, when the the same type of graft was used with a relaxing incision procedure instead, results were promising[75]. The incidence of ED seems to be higher in patients who underwent plaque excision and dermal grafting than those who underwent incision and grafting (G. Jordan, personal communication)[14]. From a technical point of view, an H-shaped incision in the plaque and placement of the graft is often all that is required after mobilizing an appropriate length of the neurovascular bundle, although plaque excision may be required when the plaque is large, thick, or calcified[8,39].

## Incisional Procedures

Recent clinical data have shown that, in many cases, plaque or tunica excision can be avoided. The creation of special relaxing incisions and inlay of a graft material can straighten the penis by expanding rather than replacing the tunica[76]. A number of studies have been described favoring incisional procedures.

Gelbard described different types of relaxing incisions leading to straightening of penile curvature. Of 93 operations performed for penile straightening, 69 involved autologous free grafts into relaxing incisions, without use of an implant, and 24 involved relaxing incisions with penile prosthesis implantation, but no grafts. When autologous grafts were used without an implant, more than 80% of the patients with dorsal or ventral bending had a good result, compared to only 17% with lateral bending. Relaxing incisions were uniformly effective in correcting penile curvature when used in conjunction with a prosthesis[77]. In an older, smaller study, the same author reported 100% success rate (no curvature recurrences) with incision of the plaque and grafting with temporalis fascia in 12 patients with a follow-up of 2 years[76].

Plaque incision and vein grafts (harvested from the deep dorsal or saphenous veins) have been reported by several authors. In 1998, Lue and El-Sakka[78] described their technique using venous patch grafting. Graft material was obtained from the lower and upper saphenous and deep dorsal veins. With the aid of a vascular stapler, several vein segments could be assembled easily into one piece to cover the defect. In most cases, an H-shaped tunical incision was adequate to release the contracture. In an outcome analysis of the above technique, El-Sakka et al. reported a 96% rate of successful penile straightening, a 13% rate of potency loss, and a 92% rate of overall satisfaction in 113 men with PD and postoperative follow-up of 18 months[74]. Using a similar technique, Metin and colleagues reported their results on 18 patients with PD-induced, dorsal penile curvature. Complete penile straightening was achieved in 16 cases. None of the patients returned to baseline preoperative angulation state in the follow-up period. Three patients reported penile shortening, but only one was concerned and showed dissatisfaction about it. Six patients had transient changes in penile sensation that resolved within 4 months[79]. In 2008, Kadioglu et al[44] assessed the outcomes of different surgical techniques used in PD surgery. In this single-center experience with 145 patients, 75 underwent simple plaque incision and IV grafting with sapheneous vein. By history, 53 (70.7%) patients were regarded as potent without any pharmacologic aid; meanwhile 22 (29.3%) of the patients were having sexual intercourse with oral or intracavernous pharmacotherapy. Seventy of 75 patients were regularly seen with a mean follow-up of 41.7–35.1 months. Penile curvature was completely straightened in 53 (75.7%) patients, whereas 12.8 and 11.4% had residual curvature less than 20 degrees and more than 20 degrees, respectively. Sixty-one (87.1%) patients preserved their potency, whereas six (8.6%) of them noticed *de novo* ED.

Long-term results of venous grafting for PD showing patient satisfaction rates are available. In a study reporting 5-year data for 50 patients with a mean age of 49 years, Montorsi et al. extensively evaluated subjective and objective parameters and yielded a rate of 88% for satisfactory correction of curvature, but there was deterioration in orgasmic and erectile function of 41 and 34%, respectively, as measured by the IIEF and Erectile Dysfunction Index of Treatment Satisfaction[80]. Similarly, Kalsi et al. evaluated 113 patients who had correction by plaque incision and saphenous vein grafting[81]. Of the 51 patients with 5 years of follow-up, the penis remained completely straight in 80%, but the incidence of ED increased to 22.5% and penile shortening of greater than 1 cm to 35%. Adeniyi et al. reported results with the Lue procedure in 51 patients with a mean follow-up of 16 months[82]. Ninety-two percent reported a satisfactory functional outcome and 82% had complete straightening of their penises. However, 35% reported penile shortening, 12% had difficulty with intercourse, and 8% developed ED after surgery.

Knoll recently reported the use of porcine jejunal submucosal tissue as a graft material[83]. Surgisis® ES, derived from porcine small intestine, was immersed in normal saline and was used to graft the tunical deficit after plaque incision using a subcoronal incisional approach in 162 patients. Penile curvature was achieved in 91% of the patients. At a mean follow-up of 38 months, 79% of the patients were fully potent, with 21% requiring assistance. No reports of intraoperative penile shortening, long-term pain, infection, bulging at the graft site, or evidence of a local immunogenic rejection reaction have been noted.

Kovac and Brock conducted a retrospective analysis of 36 patients, evaluating the surgical outcomes and patient satisfaction after dermal, pericardial (Tutoplast®), and four-layer small intestinal submucosal (Stratasis®) grafting following PD plaque incision[61]. Overall patient follow-up time was 673 ± 98 days.

Self-reported resolution of penile curvature was noted in 60% of dermal, 100% of Tutoplast, and 76.9% of Stratasis graft recipients. Stratasis patients maintained presurgery length (54%) and rigidity (77%), more so than dermal (30 and 60%) and Tutoplast (23 and 39%) patients. Assessment of ED using the IIEF-5 captured significant improvements in patients receiving Stratasis grafts.

Egydio et al.[75] described the geometric principles applied to a single incision model of "tripod-shaped 120-degree forks". An incomplete circumferential incision, forked at the extremities, was made in the tunica albuginea and in the plaque at the point of maximal curvature, followed by a septal incision for the lengthening of the short side of the penis. A bovine pericardium graft was used to repair the defect in the tunica. The authors reported their results in 33 subjects with an average follow-up time of 19.4 months. All patients maintained their state of preoperative erection, with the penis corrected in 87.9% of cases. All recovered their ability to penetrate with no difficulty. Moreover, an intraoperative average increase of 2.21 cm occurred in the size of the penis. Of note, no rejection or retraction of the graft was observed.

In 2003, Schwarzer et al. treated 18 PD men with penile corporoplasty and tunica albuginea free grafts obtained from the proximal corpus cavenosum[84]. In a preliminary follow-up of 16 patients, 12 penises were straight and four had a residual curvature less than 20 degrees. Fourteen of 16 patients were satisfied with the result of penile straightening. No severe perioperative complication was noted. In a similar review, Hatzichristou et al. also used tunica albuginea free graft combined with corporoplasty in 17 patients. They reported complete penile straightening in all patients with a mean follow-up of approximately 40 months[85].

Kargi et al. reported on a relaxing incision and fascia lata grafting[86]. When this technique was used for 12 patients, penile curvature was corrected and normal erections were achieved. Additionally, all had statistically significant penile lengthening. No complication was observed after a mean follow-up of 10 months. Alternatively, Teloken et al. yielded adequate penile straightening in six out of seven PD patients, utilizing plaque incision and grafting with the patient's own proximal harvested crura with a perineal incision[87].

Cormio et al recently evaluated the efficacy, safety, and reproducibility of plaque incision and buccal mucosa grafting in 15 men followed prospectively[88]. Follow-up visits were scheduled at 1, 3, 6, and 12 months postoperatively, then yearly. No complications were reported. All patients resumed unassisted intercourse 1 month after surgery. The 3-month postoperative evaluation showed 100% penile straightening, 1.8-cm mean increase in length of affected side, no curvature recurrence or *de novo* ED, 1.6 mean increase in IIEF-5 score, and patient and partner satisfaction of 93.3 and 100%, respectively.

The use of plaque incision and polytetrafluoroethylene (Gore-Tex) graft has been reported. Ganabathi et al.[89] applied such a technique on 16 PD patients. On a mean follow-up of 47 months, all 16 men had excellent erections with satisfactory intercourse. It should be pointed out that Gore-Tex is considered to be a thick graft material, difficult during reoperation secondary to significant fibrosis, and is easily palpated by many patients[20].

In order to address the issue of complex PD deformity, Perovic and Djordjevic introduced the concept of ''penile disassembly''[90]. Briefly, in this technique, the corporal bodies are separated from the glans, neurovascular bundle, and urethra followed by plaque incision and a dermal or vein graft interposition. Despite its complexity, the authors reported an 87% (40 of 46) rate of completely straightened penises. The remaining patients had minimal deformity. No significant complication was reported.

Finally, Bella et al.[91] recently advocated a novel approach of minimally invasive intracorporeal incision for discrete plaques less than 2 cm, without the need for tunical graft coverage. Following limited mobilization of the neurovascular bundle, a 1-cm incision lateral to the plaque allows for the introduction of a triangle-shaped scalpel designed for endoscopic carpal tunnel release into the corpora. Multiple intracorporeal incisions are made, preserving the outer layer of the tunica. The small corporotomy, Buck's fascia, and skin are then reapproximated. In 23 patients with a mean follow-up of 25 months, correction of the deformity was successful in all but two patients, with 80% of patients reporting erections sufficient for intercourse and treatment satisfaction of 85%.

## Vascularized Flaps

Krishnamurti was the first to popularize the use of the vascularized flap for defect reconstruction in PD to minimize the possible graft contracture experienced with many free grafts[92]. He published his experience over 4 years in 17 patients in which a dermabraded penile dermis was raised on its vascular fascial pedicle and used to close the defect in the tunica albuginea consequent on plaque incision[93]. The technique was feasible in both circumcised and uncircumcised patients. Five patients out of 17 received concomitant penile implant. At follow-up, all patients reported satisfactory sex lives and pain-free straight penises. There was no reported postoperative contracture or impotence. The author feels that the dermal flap is superior to free grafts based on number of observations as shown in Table 3.

**TABLE 3**
**Advantages of Penile Dermal Flap in PD Reconstructive Surgery[93]**

- Good elasticity and strength
- Avoids contracture
- Avoids penile shortening
- No extra incisions (locally available)
- Single operating team
- Employed in both circumcised and uncircumcised phallus
- Can cover large/multiple defects
- No inclusion cyst formation
- High patient satisfaction rate
- Low postoperative impotence

Helal and colleagues[94] discussed the surgical technique for the tunica vaginalis flap in the management of disabling PD, after excision of the plaque in 12 patients. Through a scrotal incision, the most dependent part of the tunica was dissected from the testicle and epididymis. The flap measured at least 4 cm in width and its upper extremity was left attached to the cremasteric muscle, which was then brought underneath a groin skin bridge to cover the penile defect. They reported a 58.3% rate of erections sufficient for vaginal penetration; however, 41.7% of the patients experienced postoperative curvature and were unable to perform sexually, 8.3% had glanular hypoesthesia, and 8.3% were noted to have venous leakage.

Knoll et al reported a novel technique that can be implemented in cases of extensive penile fibrosis, including severe PD to maximize functional penile length[95]. The authors described 11 patients with severe penile fibrosis who underwent penile prosthesis implantation with a modified suprapubic V-Y advancement flap and lower abdominal tissue debulking. The accompanying flap procedures allowed these patients to gain an additional 3.5–6.5 cm functional length. After a mean follow-up of 12 months, all patients had a functional device. No infected prosthesis was reported and no additional flap procedure was required.

The utilization of flaps in PD penile reconstruction has been examined in animal models, with variable results. Stefanovic et al. performed an experimental study on 24 male rats, evaluating the advantages of vascularized vs. nonvascularized temporoparietal free fascia transfer as an improved form of penile tunica albuginea replacement[96]. All 24 rats developed straight corpora cavernosa during erection. The flap was successful in each case, providing satisfactory morphologic appearance with minimal bulk. However, when histological assessment was applied, secondary degenerative changes were identified in all penises with nonvascularized fascial transfer, and one-third of penises with vascularized temporoparietal fascia free transfer arguing in favor of the latter.

A comparative study was carried out by Brannigan et al that looked at the histological and cavernosometric changes in the dog penis as a result of the placement of patch material (superficial dorsal penile vein, silicone fabric, and dermabraded preputial flap)[97]. Eleven mongrel dogs were divided into three groups, each receiving one of the above patch materials. The only difference among the cavernosometric parameters (preop vs. postop) was a higher initial pressure in the dermabraded preputial flap group postoperatively. The flap patch group also had mild-moderate fibrosis, with focal loss of the cavernosal space underlying the flap. Those dogs receiving a silicone patch had moderate fibrosis, with a fibrous sheath of compressed histiocytes and fibroblasts enveloping the graft site. The dogs treated with vein patch had moderate fibrosis, with apparent reformation of the tunica albuginea over the patch site. The normal venous architecture of the graft was no longer recognizable.

## PENILE PROSTHESIS IMPLANTATION

When patients with PD have both penile deformity and ED refractory to oral and/or intracavernosal pharmacotherapy, or are not candidates for these modalities, penile prosthesis implantation with or without additional straightening maneuvers is a well-accepted option[5,8,20]. With the continued advances in the medical treatment of ED, the number of candidates for penile prosthesis insertion is decreasing. Once indicated, in the majority of patients with mild to moderate curvatures, the implantation of a penile prosthesis alone is sufficient to bring about full penile straightening. However, if angulations greater than 20 degrees persist, then additional maneuvers are needed to create a straight, functional penis[14,24]. The associated procedures include modeling, Nesbit/plication, or incision/excision and grafting for penile rectification and/or correction of constrictive lesions[98] (Table 4).

**TABLE 4**
**Indications for Modeling vs. Plication/Incision/Excision Procedures during Penile Prosthesis Implantation in PD**

| Penile Prosthesis Insertion Alone | Modeling | Plication/Incision/Excision |
|---|---|---|
| Mild to moderate curvatures[20,99] | Inadequate straightening after modeling[25,100,101] | Failure of modeling to achieve straightening[24,102] |
| Angulations less than 20 degrees[14] | Angulations 20–60 degrees[14,34] | Major penile size loss (except for plication)[2,98,103] |
| | | Severe narrowing (bottlenecking/hourglass deformity)[98] |
| | | Unilateral cavitation[14,24] |
| | | Curvatures greater than 60–90 degrees[14,20,24] |
| | | Ventral plaques or large dorsal plaques (>4 cm)[104] |

In 1994, Wilson and Delk introduced the concept of modeling the penis over a penile prosthesis, in their case an inflatable device[101] (Fig. 3). The technique involves manual and forcible bending of the shaft in the direction opposite to the curvature with the prosthesis inflated. Before this is performed, the tubing from the cylinders must be clamped with a rubber-shod clamp to protect the pumping mechanism of the prosthesis. A characteristic crackling or snapping sound is usually heard with successful attempts. Of the 138 patients treated, 118 (86%) achieved a straight, rigid erection. Modeling led to urethral perforation in

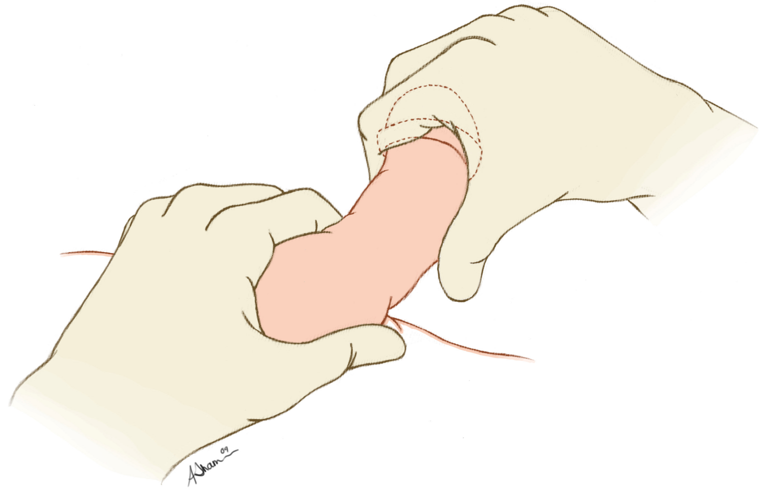

**FIGURE 3.** Operative modeling of the penis over a penile prosthesis. Manual and forcible bending of the shaft in the direction opposite to the curvature with the prosthesis inflated.

four patients (3%), whereas infection of the prosthesis occurred postoperatively in four patients (3%). At a mean follow-up of 32 months, 124 patients (90%) were using the penile prostheses, and none reported penile shortening or impairment of glandular sensation. Manual modeling has allowed some patients with PD to circumvent the need for grafting procedures[20].

In a retrospective study, Ghanem et al evaluated the surgical outcome with malleable penile implants without performing any manipulation of the plaque in 20 PD men[105]. Over a follow-up of 1 year, the authors reported an 80% success. No operative or postoperative complications occurred and no reoperations were needed. The insertion of malleable implant without additional plaque surgery was also reported by Montorsi et al., who assessed the long-term acceptance and satisfaction, but had less promising results[106]. A total of 48 patients and 29 partners were reassessed at a follow-up of at least 60 months. Only 23 patients (48%) and 12 partners (40%) were totally satisfied with the long-term functional result and would repeat the same operation. Of the partners, 17 (60%) were dissatisfied due to poor penile girth, sensation of a cold glans penis, sensation of unnatural intercourse, and dyspareunia. Eight patients (16%) chose to substitute the malleable implant with a three-component inflatable prosthesis. Although it is easier to insert, the semi-rigid implant is less preferred to the inflatable prosthesis because it is associated with poor cosmetic result, inability to mimic natural erection, decreased sensitivity or coldness in the glans, and poor concealment[26].

In 1996, Montague et al. reported a comparison between the ability of the CX and Ultrex cylinders to straighten the penis in a total of 72 patients receiving a three-piece, AMS 700 series, inflatable penile prosthesis for their PD-induced ED[107]. All 34 patients receiving the girth-expanding CX cylinders achieved complete penile straightening with cylinder inflation and bending alone. In 10 of 38 patients receiving the girth- and length-expanding Ultrex cylinders, complete straightening with cylinder inflation and bending could not be achieved, and simultaneous corporoplasty was necessary. Long-term results with the Ultrex prosthesis are not satisfactory and this device is currently not recommended in PD cases[20].

The Montorsi group continued to contribute to this area by examining the role of multiple relaxing incisions of the tunica albuginea with subsequent placement of a three-piece, inflatable penile implant (AMS CX) in 10 patients evaluated prospectively[108]. At the 6-month follow-up, all IIEF domains were significantly improved compared with preoperative values. Average penile length was increased 2.3 and 3 cm, while flaccid and erect, respectively, compared with before surgery. Complete penile straightening was achieved in nine of 10 cases (90%). Furthermore, an older study by Montorsi et al. also used the

AMS 700CX penile prosthesis for the treatment of 33 PD patients, and reported 79 and 75% patient and partner satisfaction rates, respectively[109]. Similarly, Carson[72] described the insertion of the AMS 700CX inflatable penile prostheses in 30 men with PD and ED, and found that 93% of patients had complete penile straightening.

Usta et al.[68] reviewed patient and partner satisfaction and long-term results of 42 men treated with either the AMS 700CX or Mentor Alpha-1 inflatable penile prosthesis with modeling or plaque surgery. The success rate in patients who underwent inflatable penile prosthesis implantation with manual modeling or pericardial grafting in the long-term was reported as 88%. Overall patient and partner satisfaction rates were 83.6 and 76.9%, respectively.

In 2004, Mulhall et al. discussed the treatment of 36 men with combined PD and ED using the Mentor Alpha-1 prosthesis, and assessed the need for intraoperative adjuvant maneuvers[110]. In all men with preoperative curvature of less than 30 degrees, placement of a prosthesis and full inflation was sufficient to achieve a straight penis. In men with a curvature greater than 45 degrees, 86% required plaque incision to result in a straight penis. Only 5.5% required a graft, and these men had curvature of greater than 60 degrees.

In a small series of five patients, Rahman et al.[111] reported a simplified technique that combined penile plication and three-piece, inflatable penile prosthesis placement to correct severe penile deformity and curvature. In each case, penile prosthesis placement was insufficient to correct the malformation and the addition of usually one to two pairs of plication sutures was required. The curvature in all patients was corrected with this technique. To date, no patients have reported any complications with follow-up ranging from 3 to 36 months.

Kadioglu et al.[25] recently showed that a penile prosthesis insertion, either alone or combined with penile modeling, can achieve penile straightening in up to 75–85% of men, using knowledge obtained from the contemporary literature. An inflatable prosthesis is preferred over a semi-rigid prosthesis or tunica-expanding devices. If penile deformity persists, simple plaque incision without grafting can increase the success rate to 95%. Incision of the plaque with grafting over a prosthesis is usually reserved for patients with curvature of more than 60 degrees or a dorsal plaque that is longer than 4 cm.

## OPTIMIZING SURGICAL OUTCOMES

Tunica-preserving procedures, such as Nesbit or tunical plication, have had reasonable success; however, they are associated with penile length loss. Plaque incision/excision with graft replacement may also lead to increased penile foreshortening due to scar formation, graft contracture, and curvature recurrence. Very few studies have been published that address the issue of shortening prevention following PD penile reconstruction.

Lue and El-Sakka[112] evaluated the results of chronic intermittent stretching with a vacuum erection device following circumferential tunical incision and circular venous grafting carried out in four patients. Subjects were instructed to stretch the penis with a vacuum device for 30 min daily for 6 months, starting 1 month after surgery. Penile length was 1 in. longer in the patient with only a 6-month follow-up who did not use the vacuum device, and 2 in. longer in the other three patients who used the device. Two patients who continued to use the vacuum device almost daily gained 3 in. at 18-month follow-up.

Tissue-expansion therapy has been utilized in medicine for decades, starting with Ilizarov in 1969[113]. It is well documented that gradual expansion of tissue results in the formation of new bone and connective tissue[114]. Recently, interest has been shown in penile traction devices for the treatment of PD, relying on the principle of tissue expansion over time[115]. Levine and Newell[116] described the device, which works by holding the penis in a cradle and subjecting it to gentle stretching; the tension being provided by small metal extensions that are added to the cradle frame in order to provide traction against internal springs (Fig. 4). Patients require minimal education for the device, but compliance with the extended daily treatment procedure is critical for significant curvature change.

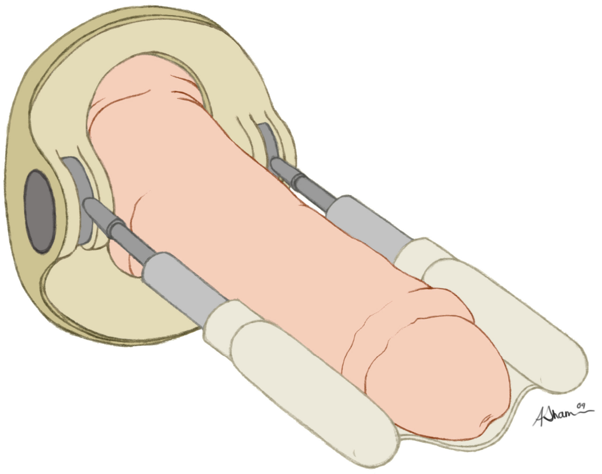

**FIGURE 4.** Penile traction device.

Levine et al. reported the first institutional review board–approved study to examine the concept of traction therapy on the penis for men with PD as a nonsurgical treatment in a single-center pilot study[117]. Using the FastSize Penile Extender, 10 patients had traction applied as the only treatment for 2–8 h/day for 6 months. Subjectively, all men noted reduced curvature estimated at 10–40 degrees, increased penile length (1–2.5 cm), and enhanced girth in areas of indentation or narrowing. Objective measures demonstrated reduced curvature in all men from 10–45 degrees. Stretched flaccid penile length increased 0.5–2.0 cm and erect girth increased 0.5–1.0 cm, with correction of hinge effect in four out of four men. IIEF-Erectile Function Domain increased from 18.3–23.6 for the group. There were no adverse events.

During the AUA 2008 Annual Meeting, Gontero et al.[118] presented results from a phase 2 prospective study, which looked at 15 men who had no or mild ED and a penile curvature of less than 50 degrees, who were instructed to wear penile extenders (Andropenis®; Andromedical, Madrid, Spain) for at least 4 h/day for 6 months. At the 6-month follow-up, the mean curve had reduced from 31 to 27 degrees and mean stretched penile length had increased 1.4 cm. The benefit was maintained at 12-months follow-up. Although the treatment effect was negligible, overall treatment satisfaction was surprisingly positive. The authors attributed this satisfaction to the length recovered through use of the penile traction device.

Few abstracts have been presented that evaluate the use of a mechanical device to stretch the penis following PD reconstructive surgery. Moncada-Iribarren et al.[119] described their analysis that involved 28 men, with a mean age of 58, who either underwent plaque incision with grafting (eight subjects) or plication of the tunica albuginea (20 subjects). A total of 14 patients were treated with a postoperative penile extender (Andropenis device) daily (8–12 h of continuous stretching ranging from 900 to 1200 g). Sustained treatment for 4 months with the stretching device provided an increase from 1 to 4 cm and an increase in girth from 0.5 to 1.5 cm. Comparing the results of the SF-36 health survey, a significant difference could be observed between both groups ($p < 0.001$). The use of the device was generally well tolerated; only two patients had moderate penile pain. No other complications were recorded. Moncada-Iribarren recently described another series of 40 men[120]. Twelve patients were submitted to a grafting procedure, while the rest (n = 28) underwent a plication technique. Twenty consecutive patients were treated with a penile extender device (Andropenis), while the previous 20 served as a control group. The extender was applied (2–3 weeks after surgery) for 8–12 h daily for at least 4 months. Treatment with the device produced a length increase ranging from 1 to 3 cm; this increase was proportional to the number of hours per month the patient used the extender. There were significant differences in several of the SF-36 parameters in the patients using the device when compared to those not using the extender ($p < 0.001$).

Four patients had moderate pain, requiring reduction of the device application time. In another study with a smaller population, Pozza et al. described 10 patients who were treated with plaque removal and autologous venous patch[121]. Five of the 10 patients were started on penile extender (Andropenis) from day 10, for an average of 2 h at morning/afternoon/evening, and were compared to the remaining five men who did not use the device. At 3-month follow-up, all the five patients who used the device showed no reduction in size, no curvature of the penile shaft, and an adequate penetrative activity. Among the remaining patients, two developed progressive curvature and one had patch retraction. Of note, all 10 patients received a vascular "rehabilitation" therapy with sildenafil 25 mg in the evening on alternate days, for 20 days starting at day 7.

## ROLE OF PHOSPHODIESTERASE 5 (PDE5) INHIBITORS IN PD

As we mentioned earlier, a significant number of patients with PD have ED. The ED may be secondary to PD or might be occurring simultaneously[8]. The causes of ED in PD include penile vascular disease (arterial and/or venous), penile deformity that makes sexual activity difficult, flail penis that causes a segmentally absent tumescence, and performance anxiety related to the presence of the physical abnormality[122]. Furthermore, ED may predispose to the development of PD in the susceptible individual, since a decrease in the rigidity of the penis may set up an opportunity for the "at-risk" penis to undergo repetitive bending, which may result in a "metal fatigue–like" fracture within the tunica albuginea, leading to the activation of the disordered healing wound process[6]. A concern in regard to surgical treatment of PD is the postoperative incidence of ED. Choi et al. nicely studied the incidence of ED in 56 (mean age of 57 years) patients who underwent incision and grafting for PD[123]. Mean baseline and postoperative IIEF scores were 23 and 17, respectively; 46% of patients had a decrease of six points or more in IIEF score. The authors reported that the following were considered predictors of postoperative ED: preoperative curve of greater than 60 degrees, use of the Egydio geometrical incision (vs. the standard H-type incision), patient age greater than 55 years, and baseline venous leak.

In the past, many PD patients with associated ED underwent a placement of a penile prosthesis. However, with the advances in medical treatment, most authorities believe that prosthesis implantation should be reserved as the final treatment modality in men with concurrent severe ED unresponsive to pharmacological treatment[24]. Many men with PD have avoided sexual contact since the onset of their disease because of the extreme curvature of their penis and uncertainty of their erectile capability. Therefore, a home trial of PDE5 inhibitors with a subsequent follow-up report in the clinic may help in deciding if surgical intervention is needed, as well as selecting the procedure of choice[100]. Nevertheless, physicians and patients have been reluctant to use PDE5 inhibitors for ED in patients with PD because all three FDA-approved PDE5 inhibitors (sildenafil, vardenafil, and tadalafil) have cautionary notes in their package inserts regarding use of these agents for men with anatomical deformation of the penis (such as angulation, cavernosal fibrosis, or PD)[124]. This warning was required since men presenting with ED in association with PD were not specifically evaluated during the clinical trials prior to FDA approval[34].

Levine and Latchamsetty published the first report on the effectiveness of using a PDE5 inhibitor as a potential first-line agent for treating ED associated with PD[124]. In this open-label retrospective study, 73 men were given a trial of sildenafil and were allowed to titrate to 100 mg. Treatment satisfaction was determined by interview in the office as well as using a modified treatment satisfaction (EDITS) questionnaire[125]. Overall, 70.8% were either very or somewhat satisfied with their erectile response. There was no reported penile injury during coitus while taking sildenafil, nor was any worsening of deformity noted during the treatment period.

Mulhall et al. developed a surgical algorithm for men with combined PD and ED based on response to erectogenic pharmacotherapy[21]. In this prospective, nonrandomized analysis, 62 patients were initially treated with sildenafil. Successful response was defined as the development of a penetration rigidity erection by patient self-report. Patients who failed to respond to sildenafil underwent intracavernosal injection therapy (ICI) before a decision was made regarding surgical therapy. Nonresponders to erectogenic pharmacotherapy were advised to undergo penile prosthetic surgery. Responders to erectogenic therapy were considered candidates for either corporoplasty or plaque incision and grafting. Eighty-one percent of the patients responded to erectogenic pharmacotherapy and thus avoided penile implant surgery. The authors reported excellent postoperative IIEF erectile function and satisfaction scores for corporoplasty and implant patients; however, plaque incision and grafting patients had poor functional and satisfaction outcomes, indicating that it is perhaps a poor option for men with combined disease.

PDE5 inhibitors have also been shown, in an *in vitro* model, to exert a potential antifibrotic affect against a Peyronie's-like scar[34]. Two rat models for PD have been developed in which a PD-like plaque in the tunica albuginea is elicited in rats by injection with either TGF-β1 or fibrin into the tunica albuginea[126,127]. The development of the plaque in both rat models is accompanied by expression of the inducible form of nitric oxide synthase (iNOS), after which nitric oxide (NO) and its down-stream product, cGMP, are produced continuously[128]. Long-term inhibition of iNOS activity, presumably by blocking NO and cGMP-mediated effects triggered by iNOS expression, exacerbates tissue fibrosis through an increase in: (a) collagen synthesis, (b) levels of reactive oxygen species (ROS), and (c) the differentiation of fibroblasts into myofibroblasts[129]. iNOS has also been found to be expressed in the fibrotic plaque of PD from human tissues[129]. It is thought that iNOS, and hence NO, acts as an antifibrotic defense mechanism since, in the rat model, long-term administration of an iNOS inhibitor enhances fibrosis of the PD-like plaque and induces general corporal fibrosis[130]. Davila et al.[131] investigated the antifibrotic role of iNOS by determining whether a plasmid-expressing iNOS (piNOS) injected into a PD-like plaque can induce regression of the plaque. A PD-like plaque was induced with fibrin in the penile tunica albuginea of mice and then injected with a luciferase-expressing plasmid (pLuc), either alone or with piNOS. Rats were treated with either piNOS, an empty control plasmid (pC), or saline. Other groups were treated with pC or piNOS in the absence of fibrin. Luciferase reporter expression was restricted to the penis, peaked at 3 days after injection, but continued for at least 3 weeks. In rats receiving piNOS, iNOS expression also peaked at 3 days, but expression decreased at the end of treatment, when a considerable reduction of plaque size occurred. The piNOS gene transfer regressed the PD plaque and expression of profibrotic factors. cGMP also seems to have antifibrotic actions; it inhibits collagen synthesis and myofibroblast differentiation in cell cultures from PD plaques and normal tunica albuginea[132]. Valente et al.[129] showed that L-arginine, which stimulates NOS activity, and hence NO synthesis, and PDE inhibitors, which increase the levels of cGMP and/or cAMP, can inhibit collagen synthesis and induce fibroblast/myofibroblast apoptosis, thus acting as antifibrotic agents. In PD fibroblast cultures, the same authors noticed that pentoxifylline (nonspecific cAMP-PDE inhibitor) increased cAMP levels without affecting cGMP levels, whereas sildenafil (PDE5A inhibitor) raised cGMP levels. The expression of variants of PDE5 and PDE4 have previously been demonstrated in normal human and rat tunica albuginea and tunica albuginea obtained from PD plaque tissue, as well as in their respective fibroblast cultures[129]. Elegant experiments by the UCLA lab group demonstrated that the administration of sildenafil in drinking water for 45 days in rats with a PD-like plaque induced by TGF-β1 resulted in an 80–95% reduction in both plaque size and collagen/fibroblast ratio[129]. Also, sildenafil was found to stimulate fibroblast apoptosis within the tunica albuginea. The authors hypothesized that increasing cGMP in target tissues prevents fibrosis.

The same group, in a similar rat PD model, explored the role of vardenafil administered at lower doses and different regimens[126]. Vardenafil was given to male rats either continuously in drinking water or as an oral instillation once a day, at low and intermediate doses, starting from the moment of TGF-β1 injection, for 45 days (early/preventive treatment). Other groups received the two doses of vardenafil only in the drinking water, starting with a well-formed plaque, for 42 days (late/therapeutic administration). The investigators found that vardenafil at the intermediate dose in the preventive treatment, both in the continuous and the once-a-day treatments, significantly reduced the collagen/smooth muscle by 80%, collagen III/I ratios by 50%, and the number of myofibroblasts and TGF-β1+ cells by 65–70%, in the PD-like plaque as compared to the similar groups without vardenafil, and selectively increased the apoptotic index in the PD-like plaque. It was concluded from this study that long-term vardenafil treatment slows down and reverses the early stages of PD-like plaque in the rat and may help to diminish more advanced plaques.

Kovanecz et al. also explored the possible antifibrotic effect of tadalafil[133]. The study involved male rats (10 per group) that either had a sham operation, unilateral cavernosal nerve resection (CNR), or bilateral CNR, and were left untreated or given 5 mg/kg per day of tadalafil retrolingually. Following 45 days of treatment, tadalafil normalized the low response to papaverine and high drop rate in the intracavernosal pressure measured by cavernosometry after CNR compared with sham-operated rats. Tadalafil was also found to normalize the increase in penile shaft collagen content, and the reduction in corporal smooth muscle cell (SMC) content, SMC/collagen, and replication index, and improved the lower collagen III/I ratio and the increase in apoptotic index, caused by CNR, compared with sham operation. Although the PDE5 inhibitor antifibrotic protective effect has been shown in patients after radical retropubic prostatectomy[134], there has been no published human data that examines the role of PDE5 inhibitors in dissolving PD plaque in a clinical setting. At our center, we have anecdotally noticed some subjective and objective improvement in PD plaque in men treated with a small daily dose of tadalafil. These early preliminary observations may broaden the clinical application for oral PDE5 inhibitors of the future; however, a randomized controlled trial is warranted to define the true efficacy of this approach.

Finally, Levine et al. examined the concept of "sildenafil citrate rehabilitation" to be used in the postoperative patient after grafting for PD[135]. The goal of rehabilitation is to enhance nocturnal erections to nourish the graft and prevent fibrosis in the healing cavernosal tissues[34]. This noncontrolled trial compared men who received sildenafil 25 mg nightly for 40 nights beginning 1 week after surgery vs. those who did not receive it[135]. For those enrolled in the rehabilitation arm, seven out of 26 (26%) developed ED in comparison to four out of 11 patients (36%) who developed diminished rigidity when not subjected to the protocol. Although not statistically significant, there was a trend towards decreased ED in those receiving sildenafil. Obviously, further studies are needed to validate this interesting concept.

## FUTURE DIRECTIONS

PD continues to generate a large body of research in order to prevent or delay surgical intervention, as well as decreasing surgical complications and improving outcomes. When penile reconstruction is indicated, the PD plaque is often excised or incised, and the resultant defect is then covered with a patch graft. These procedures can be associated with extensive mobilization of the dorsal neurovascular bundle and large tunical defects, which together can impact postoperative erectile function. To address this issue, Fournier at al. described a procedure to ablate the plaque using the carbon dioxide laser, which allows one to remove all palpable evidence of plaque, while at the same time avoiding the creation of a large defect in the tunica albuginea that requires coverage with a large graft[136]. In the authors experience with 12 patients, it was possible to remove large dystrophic calcifications from within the plaque and to augment the repair in the area of greatest curvature using the deep dorsal vein as a patch graft. Similarly, Kim and McVary reported on incision and carbon dioxide laser ablation combined with a deep dorsal vein patch graft in the treatment of six men, with acceptable results[137]. Successful use of the neodymium:YAG laser has also been reported[138]. These techniques have not yet gained widespread acceptance; however, future advances may include the application of laser or other devices to modify, shrink, or destroy, rather than physically excise, the plaque.

Although a variety of tissue grafts have been used in PD surgery; no ideal graft material has been found. Tissue engineering is an interesting discipline that may greatly improve tissue graft materials for use in PD. Published techniques involved synthetic and natural biodegradable matrices alone, known as "unseeded" scaffolds, as well as "seeded" scaffolds[139]. This area was elegantly explored by Schultheiss et al., who generated a tissue-engineered type of mechanically stable graft suitable for surgical replacement of the tunica albuginea[140]. In this study, porcine fibroblasts isolated from open fascia biopsies were seeded on decellularized collagen matrices and then cultivated in a bioreactor under continuous multiaxial stress for up to 21 days. Static cultures without mechanical stress served as controls. Only the mechanically strained cultures of fibroblasts showed a homogeneous multilayer matrix infiltration and a regular cell alignment in the direction of strain axis after 7 days, as well as a *de novo* production of extracellular matrix proteins compared to the static control. A large amount of viable fibroblasts was easily obtained from small skin punch biopsies. Advances in this area are overwhelming and in the future, grafts will likely be available that are much more equivalent to the tunica albuginea, or an acellular matrix that may allow the tunica to be rebuilt, whether associated with cell culture and seeding or not[2].

Currently, grafts are sutured into place. Another avenue of research would be to examine the role of tissue-welding techniques in a hope to replace time-consuming and profibrotic suturing[141,142].

## CONCLUSIONS

PD remains a therapeutic challenge to the urologist in clinical practice. The etiology, pathophysiology, and management remain controversial. Initially, the patient should be managed conservatively and nonsurgically, particularly in the acute phase. Surgical treatment is advised for patients with disabling deformities in the chronic phase of the disease. There is no universal approach to all cases and, thus, recognition of the benefits and limitations of each surgical approach allows for the most appropriate procedure to be undertaken. Plication techniques may be applied to patients with mild to moderate deformities with satisfactory erectile function; however, penile shortening is an unavoidable consequence of these options. Men with large plaques, severe or complex deformities, or short penises may be better suited for incision or excision of the plaque and the placement of a graft material. Penile prosthesis remains a viable option in patients with pre-existing ED unresponsive to oral or intracavernous injection therapy. Manual modeling is a well-established method for correcting the majority of the persistent curvatures after implantation of the penile prosthesis. The role of vacuum erection device and penile traction devices in optimizing surgical outcomes after PD penile reconstructive surgery have been explored in limited studies, with encouraging preliminary results. Recent animal models have suggested a potential antifibrotic affect of PDE5 inhibitors against PD plaque. This is yet to be confirmed in human studies. Researchers continue to explore novel methods to best serve PD patients surgically.
